# Supplementary figures and images for: Comparative Blood Transcriptome Analysis of Semi-Natural and Controlled Environment Populations of Yangtze Finless Porpoise
Source: Animals (Basel). 2024 Jan 7;14(2):199. doi: 10.3390/ani14020199 (PMC10812818; doi:10.3390/ani14020199)

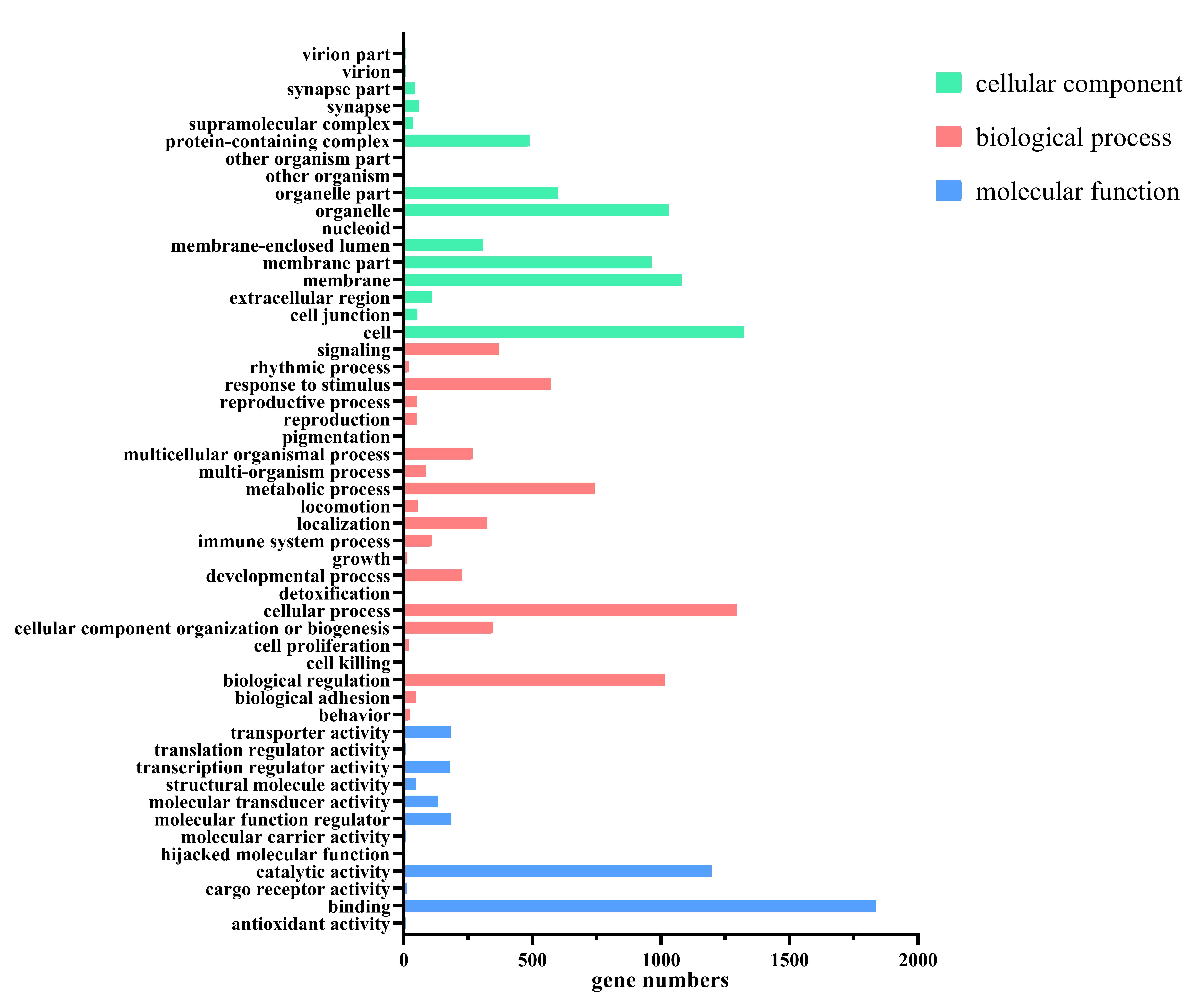

Supplement: Supplementary file 1 [file animals-14-00199-s001.zip › Supplementary Figure S1.jpg]

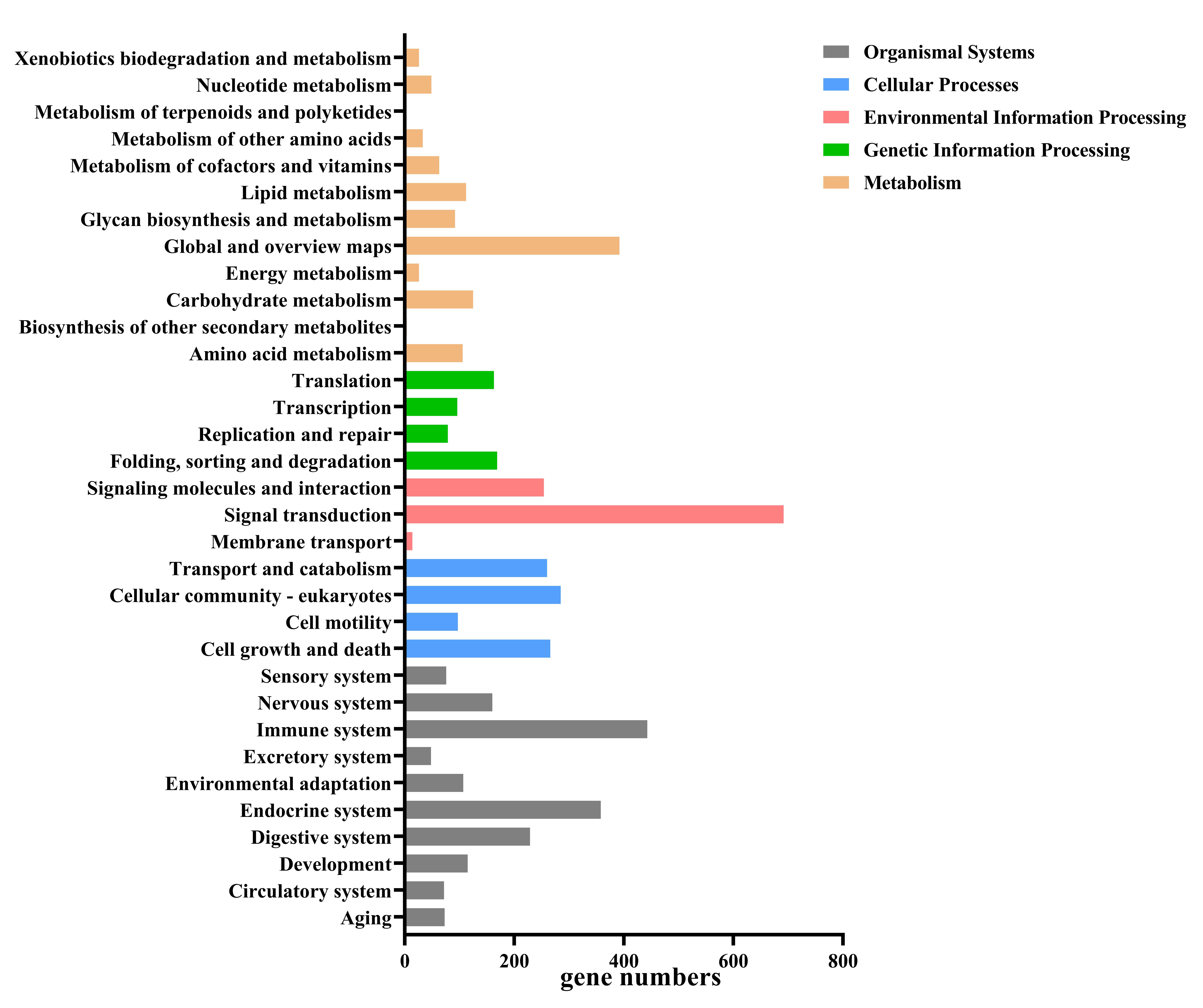

Supplement: Supplementary file 1 [file animals-14-00199-s001.zip › Supplementary Figure S2.jpg]
